# Hospital Meetings, &c.

**Published:** 1900-02-24

**Authors:** 


### HOSPITAL MEETINGS, &C.

### LONDON FEVER HOSPITAL.

Lord Balfour of Burleigh presided at the annual meeting of the London Fever Hospital, held at the Hotel Victoria on Friday last, 16th inst. The statement of accounts, presented by Mr. Hamer, the chairman of the Finance Committee, showed ordinary receipts, £11,273; extraordinary £2,910; ordinary expenditure, £10,716; and extraordinary £2,655. From the report of the committee, read by Dr. Phillips, it appeared that 700 patients were treated during 1899, 24 in excess of those in the previous year; 503 were cases of scarlet fever—126 more than last year—of whom six died; 32 were of measles, all being cured; rubeola, 33 cases, all cured; diphtheria, 89 cases, 11 of whom died. Owing to want of accommodation only two cases of enteric fever were admitted; both recovered. Thirty-eight patients were sent into the hospital under certificates of infectious disease who were not so suffering. Among the officials of the institution four nurses contracted scarlet fever, two diphtheria, one German measles; the ambulance porter also contracted scarlet fever; all recovered. Throughout the year the Governors were compelled to refuse admission to many applicants on account of want of room. The Committee of Visitors of the Prince of Wales's Fund visited the hospital during the year, and reported that "the old wards of the hospital are in an unsatisfactory condition and the sanitary blocks antiquated." The visitors were of opinion that the old part of the hospital will have to be reconstructed and brought up to date. With this the committee of the hospital had been in entire agreement for a long time past; indeed, one block of buildings had been unoccupied for some time in consequence of its dilapidated state. The report expressed the hope that in the coming year funds might be obtained for demolishing the old wing of the hospital and rebuilding it at once according to the plan adopted some years since as part of the scheme for the gradual rebuilding of the hospital. Regret was expressed at the death of Sir Richard Thorne-Thorne, one of the vice-presidents of the institution, who was formerly one of their assistant physicians. Captain W. W. Ross, an old member of the committee, had also died during the year.

The Chairman said the past year had not been one of great pressure, there being no special epidemic at a particular time, but they had treated more patients than in 1898, and not only had their daily average of 75 beds occupied been considerably larger than last year, but the duration of the occupation of the beds had been longer, and as a consequence the drain on the funds of the hospital had been more serious. This was due to the fact that in 1898 they had a larger proportion of measles, the sojourn in cases of scarlet fever and diphtheria being, of course, longer. Since 1802, when the hospital was founded, nearly 90,000 cases had been treated, mostly of scarlet fever and diphtheria. During the year they had had to refuse cases owing to lack of accommodation, but of these by far the larger number were cases of typhoid, because these could be treated in the wards of general hospitals. Their annual subscriptions, amounting to £4,546, showed a small increase of £65 on the previous year; but their donations were only £1,587—a falling off of £713. This was a matter that had been engaging the earnest attention of the committee, because they looked to this source for the reconstruction of the hospital and the building of the convalescent home. Their subscriptions were all swallowed up by working expenses, and unless they got an income from donations and legacies they could not afford to build. The Prince of Wales's Committee had commented on the state of repair in which part of the hospital was, and they were only too anxious to deal with it. But, without making any complaint, he would like to point out that during the last three years their income from donations had fallen from the average of the previous eleven years of £2,300 to £1,841, although a large special donation of £500 had come into that period. So that as compared with what they had done before the establishment of the Prince of Wales's Fund they showed an average drop of £700 per annum. He did not complain of this; he would be one of the last to attack the management of the Prince of Wales's Fund; he believed they were doing a great and good work in systematising our charities. But he did think they were entitled to point out that, taking into account what they had received from the Fund—£200 was the amount that year—but for its establishment they were entitled to believe they would be richer by nearly £2,000. And to the committee's suggestion that they should reconstruct their wards they should answer, with perfect good humour, that they would be delighted to do so if the Fund would enable them to by making them a larger grant. They had had their attention called to the offer of £25,000 recently made for the construction and maintenance of a hospital ward to be called the Annie Zuntz ward in memory of the donor's wife, and in which a full length portrait of her was to be hung, and they had notified their willingness to accept the conditions of the bequest, and hoped it might fall to them. After an appeal to the Hospital Saturday Fund to increase its grant to them since so large a proportion of their patients were drawn from the ranks of the subscribers to that Fund; and to employers whose servants received the benefits of the hospital, and whose support in many cases was far from adequate, the Chairman moved a vote of thanks to the committee.

This was carried, as was a motion for the re-election of Lord Balfour as president of the hospital, and that of the other officers and the committee, and the proceedings then terminated.

